# Impact of Size Matching Based on Donor-Recipient Height on Kidney Transplant Outcomes

**DOI:** 10.3389/ti.2022.10253

**Published:** 2022-03-18

**Authors:** Srijan Tandukar, Christine Wu, Sundaram Hariharan, Chethan Puttarajappa

**Affiliations:** ^1^ Willis-Knighton Medical Center, Shreveport, LA, United States; ^2^ University of Pittsburgh Medical Center, Pittsburgh, PA, United States

**Keywords:** kidney transplantation, size mismatch, height mismatch, weight mismatch, SRTR

## Abstract

Transplantation of kidneys from shorter donors into taller recipients may lead to suboptimal allograft survival. The effect of discrepancy in donor and recipient heights (ΔHeight) on long term transplant outcomes is not known. Adult patients ≥18 years undergoing living or deceased donor (LD or DD) kidney transplants alone from donors ≥18 years between 2000 and 2016 in the United States were included in this observational study. The cohort was divided into three groups based on ΔHeight of 5 inches as 1) Recipient < Donor (DD: 31,688, LD: 12,384), 2) Recipient = Donor (DD: 84,711, LD: 54,709), and 3) Recipient > Donor (DD: 21,741, LD: 18,753). Univariate analysis showed a higher risk of DCGL and mortality in both DD and LD (*p* < 0.001 for both). The absolute difference in graft and patient survival between the two extremes of ΔHeight was 5.7% and 5.7% for DD, and 0.4% and 1.4% for LD. On multivariate analysis, the HR of DCGL for Recipient < Donor and Recipient > Donor was 0.95 (*p* = 0.05) and 1.07 (*p* = 0.01) in DD and 0.98 (*p* = 0.55) and 1.14 (*p* < 0.001) in LD. Similarly, the corresponding HR of mortality were 0.97 (*p* = 0.07) and 1.07 (*p* = 0.003) for DD and 1.01 (*p* < 0.001) and 1.05 (*p* = 0.13) for LD. For DGF, the HR were 1.04 (*p* = 0.1) and 1.01 (*p* = 0.7) for DD and 1.07 (*p* = 0.45) and 0.89 (*p* = 0.13) for LD. Height mismatch between the donor and recipient influences kidney transplant outcomes.

## Graphical Abstract

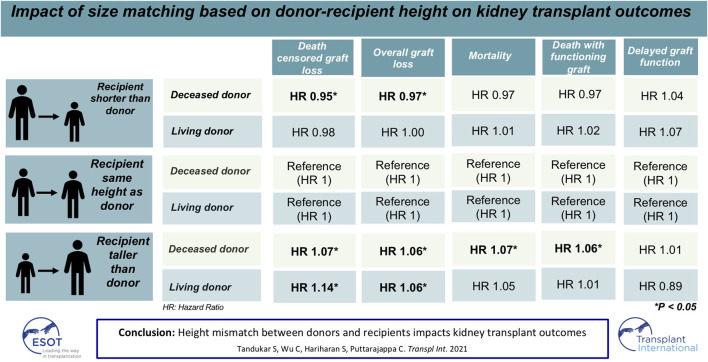

## Introduction

Differences in the size of the recipient and donor have been shown to influence kidney transplant outcomes. This difference in outcomes is postulated to be secondary to the individual’s kidney size and the number of nephrons, which is proportional to the overall size of the individual. The deficit in nephron endowment at the time of birth is permanent and does not change with the increase in demand later in life (1). There is no consensus on the anthropometric measure that best correlates with an individual’s kidney size and nephron mass. From a physiological standpoint, transplantation of a kidney with a smaller number of nephrons into a larger individual may cause the nephrons to undergo hypertrophy, hyperfiltration injury, and eventually, sclerosis, exhaustion, and fibrosis (1,2).

Size mismatch between the donor and the recipient has been studied based on differences in their weight, body mass index (BMI), and body surface area (BSA) as surrogates for kidney size and nephron mass (2,3). These studies have shown conflicting results on the effect of these discrepancies on kidney transplant outcomes. In a population based study conducted on transplant patients in the UK Transplant Registry, there was no difference in graft survival and higher mortality in patients receiving kidneys from donors with a higher weight and BMI (2). In contrast, another study from the Scientific Registry of Transplant Recipients (SRTR) based on discrepancies of BSA showed that those receiving organs from smaller sized donors based on their BSA had increased risk of graft loss, an effect that was modulated by the recipient and donor ages (3).

We hypothesized that adult height may be a more optimal measure of nephron mass in an individual. The reasons for this are 1) adult height has a strong association with birth weight and length, which are known predictors of nephron mass (4–6), 2) adult height is strongly correlated with the length of the kidney (7), 3) adult height is less prone to distortion by an individual’s lifestyle such as eating habits and physical activity, or by fluid balance in end stage renal disease (ESRD) patients, both of which may alter an individual’s weight and composite anthropometric measures such as BMI and BSA, and 4) adult height is less likely to change once an individual enters adulthood, unlike weight, BMI and BSA, which may show wide temporal fluctuations within a person’s lifespan.

This study aimed to determine whether height discrepancies in the donors and recipients predicted kidney transplant outcomes such as death censored graft survival, overall graft survival, patient survival, delayed graft function, and death with a functioning graft.

## Methods

### Patient Population

This study used data from the Scientific Registry of Transplant Recipients (SRTR). The SRTR data system includes data on all donors, wait-listed candidates, and transplant recipients in the US, submitted by the members of the Organ Procurement and Transplantation Network (OPTN). The Health Resources and Services Administration (HRSA), U.S. Department of Health and Human Services provides oversight to the activities of the OPTN and SRTR contractors.

**FIGURE 1:**
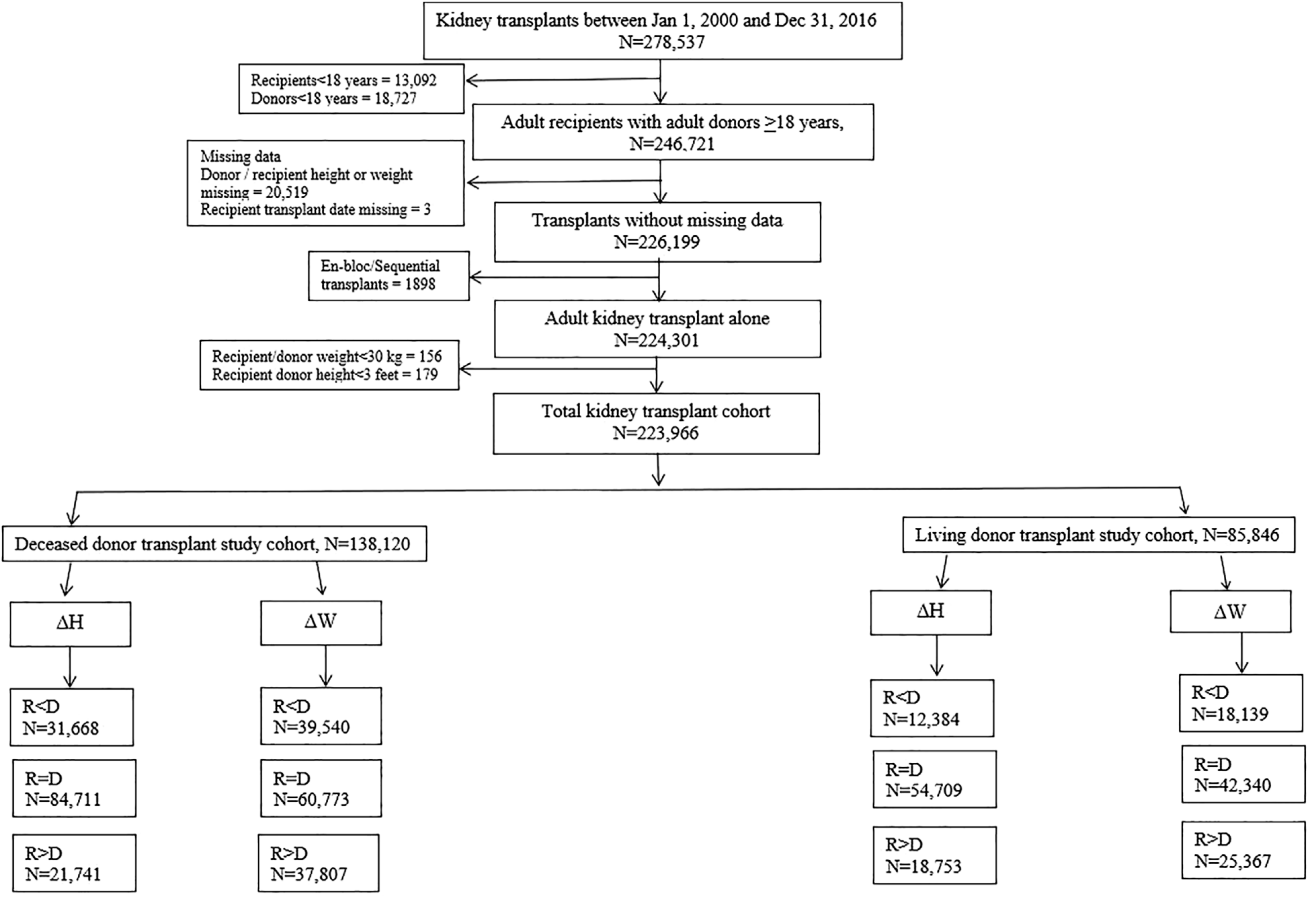
Algorithm showing derivation of patient cohorts for analysis.

Adult patients above the age of 18 years, undergoing kidney transplants alone from donors, above the age of 18 years between January 1, 2000 and December 31, 2016, were selected. The algorithm for the derivation of the study cohort is presented in Figure 1.

Baseline characteristics included the recipient and donor’s age, sex, ethnicity, dialysis vintage (for recipients), history of diabetes mellitus, hypertension, pre-emptive transplantation, height, weight, body mass index (BMI), and body surface area (BSA). BSA was calculated in m^2^ using the Mosteller formula, √ (height (cm) × weight (kg)/3600). For deceased donors, data on history of hepatitis C infection, terminal donor creatinine, cause of death, donation after circulatory death, and cold ischemia time were also recorded. Other transplant variables such as the number of HLA mismatches, peak panel reactive antibodies (PRA), acute rejection episodes, and the use of thymoglobulin for induction were also included (Tables 1, 2).

**TABLE 1:** Baseline characteristics of patients stratified by differences in donor and recipient height for deceased donor kidney transplants.

Characteristic*	Recipient >5 inches shorter than donor (R < D)	Recipient up to 5 inches taller or shorter than donor (R = D)	Recipient >5 inches taller than donor (R > D)	Overall cohort	*p*-Value
N (%)	31668 (22.9%)	84711 (61.3%)	21741 (15.7%)	138120	
**Recipient characteristics**					
Age, years	51.4 (13.8)	52.7 (12.8)	52.9 (12.3)	52.4 (13.0)	<0.001
Gender					
Male	9162 (28.9%)	55676 (65.7%)	19887 (91.5%)	84725 (61.3%)	<0.001
Race					
White	19483 (61.5%)	51504 (60.8%)	12664 (58.2%)	83651 (60.6%)	<0.001
Black	8467 (26.7%)	27093 (32%)	8265 (38%)	43825 (31.7%)	
Asian	3024 (9.5%)	4509 (5.3%)	510 (2.3%)	8043 (5.8%)	
Others	694 (2.2%)	1605 (1.9%)	302 (1.4%)	2601 (1.9%)	
Dialysis vintage, days [Median (IQR)]	1423 (839, 2191)	1449 (859, 2240)	1397 (836, 2153)	1425 (843, 2196)	0.95
Hypertension	22601 (71.4%)	61478 (72.6%)	15800 (72.7%)	99879 (72.3%)	<0.001
Diabetes mellitus	10224 (32.3%)	29765 (35.1%)	7687 (35.4%)	47676 (34.5%)	<0.001
Height, inches	63.0 (3.1)	67.4 (3.5)	71.6 (3.2)	67.1 (4.3)	<0.001
Weight, kg	71.9 (16.7)	82.3 (18.3)	92.3 (19.2)	81.5 (19.2)	<0.001
BMI, kg/m^2^	28 (5.9)	27.9 (5.5)	27.9 (5.4)	27.9 (5.6)	0.019
BSA, m^2^	1.8 (0.2)	2.0 (0.2)	2.2 (0.3)	2.0 (0.3)	<0.001
**Donor characteristics**					
Age, years	39.3 (13.9)	41.4 (13.8)	43.1 (13)	41 (13)	<0.001
Gender					
Male	28128 (88.8%)	49316 (58.2%)	5289 (24.3%)	82733 (59.9%)	<0.001
Ethnicity					
White	26635 (84.1%)	71082 (83.9%)	18135 (83.4%)	115852 (83.9%)	<0.001
Black	4297 (13.6%)	10860 (12.8%)	2617 (12%)	17774 (12.9%)	
Asian	418 (1.3%)	1999 (2.4%)	768 (3.5%)	3185 (2.3%)	
Other	318 (1.0%)	770 (0.9%)	221 (1.0%)	1309 (0.9%)	
Height, inches	71.3 (2.8)	67.6 (3.4)	63.6 (3.2)	67.8 (3.9)	<0.001
Weight, kg	90.0 (20.3)	81.9 (20)	74.9 (19.8)	82.6 (20.6)	<0.001
BMI, kg/m^2^	27.4 (5.8)	27.8 (6.4)	28.7 (7.5)	27.8 (6.5)	<0.001
BSA, m^2^	2.1 (0.3)	2.0 (0.3)	1.8 (0.3)	2.0 (0.3)	<0.001
Diabetes mellitus	1967 (6.2%)	5870 (6.9%)	1801 (8.3%)	9638 (7%)	<0.001
Hypertension	8404 (26.5%)	24930 (29.4%)	7067 (32.5%)	40401 (29.3%)	<0.001
Hepatitis C virus	651 (2.1%)	2703 (3.2%)	763 (3.5%)	4117 (3.0%)	<0.001
Terminal donor creatinine					
Cr <=1.5 mg/dl	25153 (79.4%)	70894 (83.7%)	19029 (87.5%)	115076 (83.3%)	<0.001
Cr > 1.5 mg/dl	6501 (20.5%)	13789 (16.3%)	2707 (12.5%)	22997 (16.7%)	
Cr unknown	14 (<0.1%)	28 (<0.1%)	5 (<0.1%)	47 (<0.1%)	
Donation after circulatory death	4495 (14.2%)	10506 (12.4%)	2471 (11.4%)	17472 (12.6%)	<0.001
**Transplant characteristics**					
Cold ischemia time, hours	17.9 (8.8)	17.7 (9.0)	17.6 (8.8)	17.8 (8.9)	0.021
Number of HLA mismatches					
0	3740 (11.8%)	8632 (10.2%)	1978 (9.1%)	14350 (10.4%)	<0.001
1	442 (1.4%)	1016 (1.2%)	266 (1.2%)	1724 (1.2%)	
2	1593 (5.0%)	4128 (4.9%)	1057 (4.9%)	6778 (4.9%)	
3	4536 (14.3%)	11604 (13.7%)	2991 (13.8%)	19131 (13.9%)	
4	8068 (25.5%)	21923 (25.9%)	5658 (26.0%)	35649 (25.8%)	
5	8889 (28.1%)	24823 (29.3%)	6552 (30.1%)	40264 (29.2%)	
6/Unknown mismatches	4400 (13.9%)	12585 (14.9%)	3239 (14.9%)	20224 (14.6%)	
Thymoglobulin induction	14637 (46.2%)	38803 (45.8%)	10074 (46.3%)	63514 (46.0%)	0.238
Pre-emptive transplants	440 (1.4%)	1200 (1.4%)	317 (1.5%)	1957 (1.4%)	0.804
Cause of death					
Anoxia	6813 (21.5%)	19442 (23.0%)	5709 (26.3%)	31964 (23.1%)	<0.001
Cerebrovascular/Stroke	9388 (29.6%)	32158 (38.0%)	10018 (46.1%)	51564 (37.3%)	
Head trauma	14535 (45.9%)	30596 (36.1%)	5256 (24.2%)	50387 (36.5%)	
Others	932 (2.9%)	2515 (3.0%)	758 (3.5%)	4205 (3.0%)	
Acute rejection episodes	684 (2.2%)	1814 (2.1%)	449 (2.1%)	2947 (2.1%)	0.735
Peak PRA					
0	9828 (31%)	31884 (37.6%)	9094 (41.8%)	50806 (36.8%)	<0.001
>0	21840 (69%)	52827 (62.4%)	12647 (58.2%)	87314 (63.2%)	

*Data is presented in the format of mean (standard deviation) or N (%) unless stated otherwise.

**TABLE 2:** Baseline characteristics of patients stratified by differences in donor and recipient height for living donor kidney transplants.

Characteristic	Recipient >5 inches shorter than donor (R < D)	Recipient up to 5 inches taller or shorter than donor (R = D)	Recipient more than 5 inches taller than donor (R > D)	Overall cohort	*p*-Value
N (%)	12384 (14.4%)	54709 (63.7%)	18753 (21.8%)	85846	
**Recipient characteristics**					
Age, years	47 (15)	47 (14)	48 (13)	47 (14)	<0.001
Gender					
Male	2101 (17%)	32287 (59%)	17831 (95.1%)	52219 (60.8%)	<0.001
Race					
White	9800 (79.1%)	44091 (80.6%)	15339 (81.8%)	69230 (80.6%)	<0.001
Black	1727 (13.9%)	7442 (13.6%)	2595 (13.8%)	11764 (13.7%)	
Asian	667 (5.4%)	2398 (4.4%)	589 (3.1%)	3654 (4.3%)	
Others	190 (1.5%)	778 (1.4%)	230 (1.2%)	1198 (1.4%)	
Dialysis vintage, days	789 (857)	786 (805)	792 (810)	788 (814)	0.874
Hypertension	8726 (70.5%)	39387 (72%)	13612 (72.6%)	61725 (71.9%)	<0.001
Diabetes mellitus	3302 (26.7%)	15348 (28.1%)	5531 (29.5%)	24181 (28.2%)	<0.001
Height, inches	63 (3)	67 (3)	71.3 (3)	67 (4.2)	<0.001
Weight, kg	69.4 (17)	79.4 (18.7)	91.1 (18.5)	80.5 (19.6)	<0.001
BMI, kg/m^2^	27 (6.1)	27.3 (5.6)	27.7 (5.2)	17.3 (5.6)	<0.001
BSA, m^2^	1.8 (0.2)	1.9 (0.3)	2.1 (0.2)	1.9 (0.3)	<0.001
**Donor characteristics**					
Age, years	40.3 (11.7)	41.1 (11.5)	43.3 (11.4)	41.5 (11.6)	<0.001
Gender					
Male	10466 (84.5%)	21802 (39.9%)	1387 (7.4%)	33655 (39.2%)	<0.001
Ethnicity					
White	10190 (82.3%)	45472 (83.1%)	15821 (84.4%)	71483 (83.3%)	<0.001
Black	1652 (13.3%)	6516 (11.9%)	2034 (10.8%)	10202 (11.9%)	
Asian	378 (3.1%)	2010 (3.7%)	643 (3.4%)	3031 (3.5%)	
Other	164 (1.3%)	711 (1.3%)	255 (1.4%)	1130 (1.3%)	
Height, inches	70.9 (3.1)	66.7 (3.5)	63.6 (2.6)	66.5 (3.9)	<0.001
Weight, kg	88 (15.6)	77.4 (15.4)	70.3 (13.1)	77.4 (15.8)	<0.001
BMI, kg/m^2^	27.1 (4.2)	26.9 (4.4)	26.9 (4.6)	26.9 (4.4)	<0.001
BSA, m^2^	2.1 (0.2)	1.9 (0.2)	1.8 (0.2)	1.9 (0.2)	<0.001
**Transplant characteristics**					
Number of HLA mismatches					
0	1032 (8.3%)	4925 (9%)	1254 (6.7%)	7211 (8.4%)	<0.001
1	691 (5.6%)	2907 (5.3%)	763 (4.1%)	4361 (5.1%)	
2	2033 (16.4%)	9028 (16.5%)	2486 (13.3%)	13547 (15.8%)	
3	3228 (26.1%)	14793 (27%)	4484 (23.8%)	22505 (26.2%	
4	1901 (15.4%)	8081 (14.8%)	3300 (17.6%)	13282 (15.2%)	
5	2253 (18.2%)	9486 (17.3%)	4052 (21.6%)	15791 (18.4%)	
6/Unknown mismatches	1246 (10.1%)	5489 (10.0%)	2414 (12.9%)	9149 (10.7%)	
Thymoglobulin induction	4701 (38%)	20340 (37.2%)	6918 (36.9%)	31959 (37.2%)	0.148
Pre-emptive transplants	781 (6.3%)	3082 (5.6%)	966 (5.2%)	4829 (5.6%)	<0.001
Acute rejection episodes	199 (1.6%)	918 (1.7%)	361 (1.9%)	1478 (1.7%)	0.046
Peak PRA					
0	5547 (44.8%)	27090 (49.5%)	10087 (53.8%)	42724 (49.8%)	<0.001
>0	6837 (55.2%)	27619 (50.5%)	8666 (46.2%)	43122 (50.2%)	

*Data is presented in the format of mean (standard deviation) or N (%) unless stated otherwise.

### Height and Weight Mismatch Between Donors and Recipients

The recipient and donor pairs were classified into 3 groups for DD and LD transplants separately based on height discrepancy (ΔHeight) as 1) Recipient >5 inches shorter than the donor (Recipient < Donor), 2) Recipient within 5 inches of donor’s height (Recipient = Donor), and 3) Recipient >5 inches taller than the donor (Recipient > Donor).

The recipient and donor pairs were also classified for DD and LD transplants separately into 3 groups based on weight discrepancy (ΔWeight) as 1) Recipient >15 kg lighter than the donor (Recipient < Donor), 2) Recipient within 15 kg above or below donor’s weight (Recipient = Donor), and 3) Recipient >15 kg heavier than the donor (Recipient > Donor).

A cut-off of 5 inches and 15 kg was chosen to create a well balanced sample for the groups, based on the distribution of height and weight discrepancies seen in the donor-recipient pairs (Supplementary Figures S1, S2).

### Outcome Measures

The primary outcome was death censored graft loss (DCGL). DCGL was defined as a return to permanent long-term dialysis or repeat transplantation. Secondary outcomes were patient mortality, delayed graft function (DGF), overall graft loss, and death with a functioning graft. Overall graft loss was defined as graft loss occurring either due to graft failure with a return to permanent long-term dialysis or repeat transplantation or death. Death with a functioning graft was defined as death occurring in a patient whose graft was functioning at the time of the death.

### Statistical Analysis

Continuous variables are reported as means and standard deviations for parametric, or medians and interquartile ranges for non-parametric data. Categorical variables are summarized as proportions. The baseline characteristics of the patients in different subgroups were analyzed using the Analysis of Variance (ANOVA) or Kruskal-Wallis test for continuous and chi squared test for categorical variables as appropriate. Kaplan Meier analysis was done for univariate analysis of the impact of ΔHeight on the primary outcome of DCGL and secondary outcome of patient mortality and DGF. Multiple Cox regression analysis was performed utilizing covariates known to influence transplant outcomes. For LD transplants, the covariates included were differences in recipient and donor height and weight, recipient and donor ethnicity, age, gender, history of diabetes and hypertension, number of HLA mismatches, pre-emptive transplant, history of acute rejection, induction with thymoglobulin, and peak panel reactive antibody (PRA). For DD transplants, additional covariates included were terminal donor creatinine, donor cause of death, donation after circulatory death, and history of hepatitis C virus infection. Interactions between recipient-donor height and weight differences, and between height and gender differences were evaluated to assess if these factors modified the impact of height difference on outcomes. The best model fit was determined based on Akaike Information Criterion (AIC) scores and likelihood ratio tests. All analyses were conducted in R statistical software, version 4.1.0. (R Foundation for Statistical Computing, Vienna, Austria).

## Results

### Patient Population

A total of 278,537 kidney transplants alone were performed in the US between January 1, 2000 and December 31, 2016. After excluding recipients <18 years of age (n = 13,092), donors <18 years of age (n = 18,727), patients with missing donor/recipient height or weight (n = 20,519), missing transplant date (n = 3), en-bloc or sequential kidney transplants (n = 1,898), recipient/donor weight <30 kg (n = 156) and recipient/donor height <3 feet (n = 179), the total cohort of patients remaining for analysis was 223,966. In this cohort, 138,120 were DD transplants and 85,846 were living donor transplants (Figure 1).

### Deceased Donor Cohort

The DD cohort was sub-divided into 3 categories based on ΔHeight as 1) Recipient < Donor (n = 31,668), 2) Recipient = Donor (n = 84,711), and 3) Recipient > Donor (n = 21,741) respectively (Figure 1).

The mean (SD) age of recipients and donors was 52.4 (13) and 41 (13) years respectively. There were more male recipients in the Recipient > Donor (91.5%) and Recipient = Donor (65.7%) groups and more female recipients in the Recipient < Donor (71.1%) group. The ethnic distribution in each of the ΔHeight categories (Recipient < Donor, Recipient = Donor and Recipient > Donor) corresponded to the overall transplant population in descending order of prevalence in Whites, Blacks, Asians, and other ethnic backgrounds (60.6, 31.7, 5.8, and 1.9% in recipients and 83.9, 12.9, 2.3, and 0.9% respectively in donors respectively). The terminal donor creatinine was >1.5 mg/dl in 20.5, 16.3, and 12.5% among the three height difference categories in the deceased donors. A larger proportion of donation after circulatory death (DCD) patients were in the Recipient < Donor group (14.2%), followed by Recipient = Donor (12.4%) and then Recipient > Donor (11.4%) groups. Other pertinent recipients, donor, and DD transplant-specific characteristics are summarized in Table 1.

The distribution of recipient-donor height differences at different categories of recipient weights and recipient-donor pairs with height differences greater than 10 inches stratified by weight quartiles of the recipient are shown in Supplementary Figures S3, S4.

### Living Donor Cohort

The LD cohort was sub-divided into 3 categories based on ΔHeight as 1) Recipient < Donor (n = 12,384), 2) Recipient = Donor (n = 54,709), and 3) Recipient > Donor (n = 18,753) respectively (Figure 1).

The mean (SD) age of recipients and donors were 47 (14) and 41.5 (11.6) years respectively. There were larger proportions of male recipients in the Recipient > Donor (95.1%) and Recipient = Donor (59%) groups and more female recipients in the Recipient < Donor (83%) group. The ethnic distribution followed the same pattern of prevalence as the DD cohort with Caucasian donors/recipients comprising the highest proportion followed by Black, Asian, and donors/recipients from other ethnic groups in each sub-group (Recipient < Donor, Recipient = Donor, Recipient > Donor; 80.6, 13.7, 4.3, and 1.4% in recipients and 83.3, 11.9, 3.5, and 1.3% in donors respectively). Pre-emptive transplants occurred in 6.3, 5.6, and 5.2% respectively. The use of thymoglobulin was not different across the three groups (*p* = 0.148). The largest proportion of patients were mismatched at 3 HLA antigens (26.1, 27, and 23.8% respectively). The Recipient > Donor group had a higher proportion of non-sensitized (Peak PRA 0%) patients followed by Recipient = Donor and Recipient < Donor groups (53.8, 49.5, and 44.8% respectively). Other pertinent recipients, donors, and LD transplant specific characteristics are summarized in Table 2.

### Primary Outcome

#### Death Censored Graft Loss

##### Deceased Donor Kidney Transplants

**FIGURE 2:**
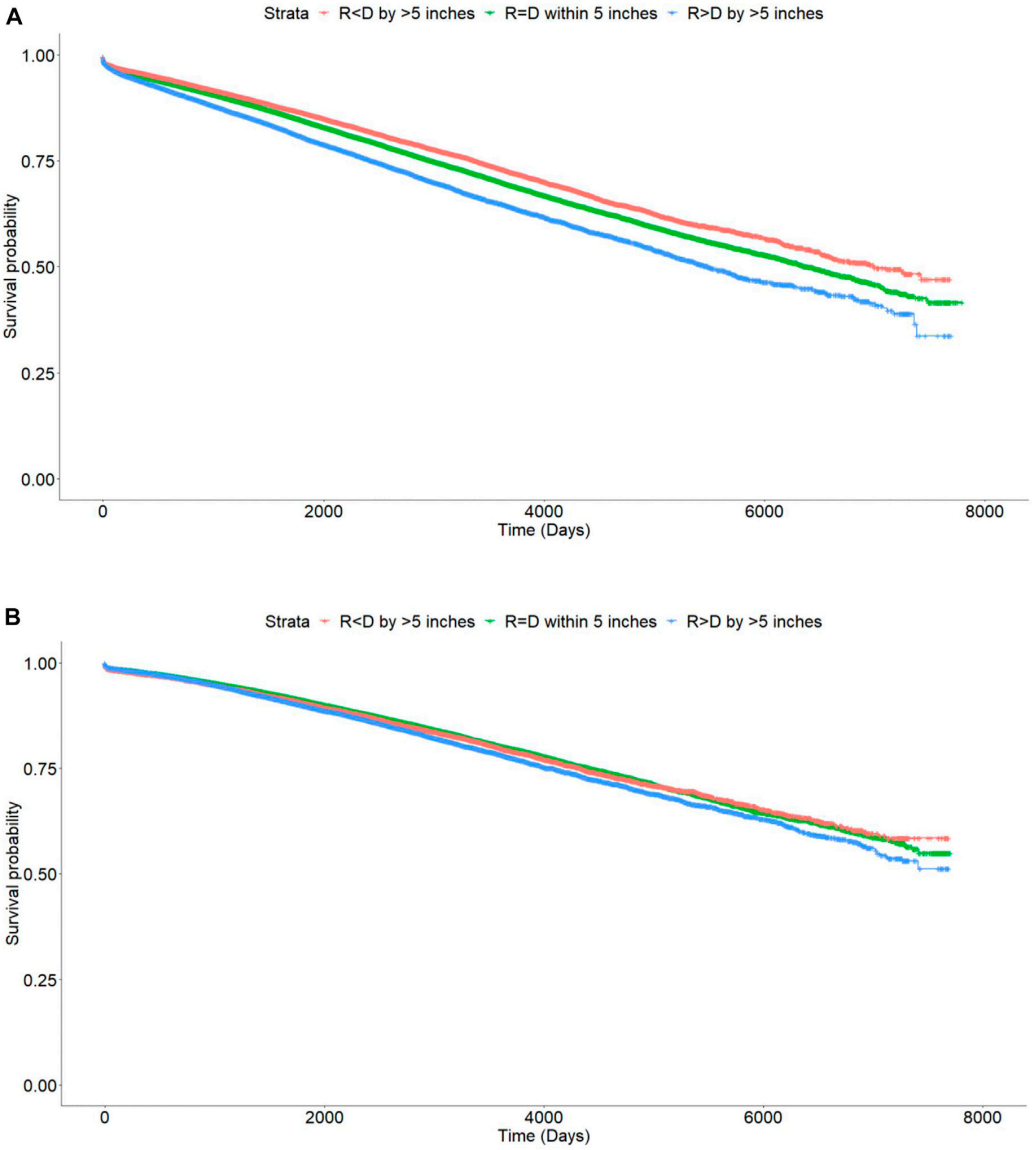
Kaplan Meier curves showing differences in death censored graft loss with incremental difference between recipient and donor heights [Height of recipient (R) shorter than (<) or taller than (>) donor (D) by more than 5 inches, or within 5 inches (=)]. **(A)** Deceased donor, *p* < 0.001. **(B)** Living donor, *p* < 0.001.

On Kaplan Meier analysis, there was a statistically significant difference in DCGL with incremental degrees of ΔHeight. The Recipient < Donor group had the lowest rates of DCGL followed by Recipient = Donor and Recipient > Donor groups (*p* < 0.001). In other words, the taller the recipient as compared to the donor, the worse the primary outcome of DCGL. The differences were more pronounced in the DD cohort compared to the LD cohort (Figure 2A).

The 1, 3, and 5 years graft survival for DD transplant recipients were 95.7, 91.5, and 87.7% for Recipient < Donor group, 94.9, 90.5, and 86.2% for Recipient = Donor group, and 93.8, 88, and 82.8% for Recipient > Donor group respectively. At last follow up, the graft survival rates were 78.8, 76.8, and 73.1% respectively. The absolute difference in graft survival rates between the two extremes of Recipient < Donor and Recipient > Donor groups was 5.7% (Table 3).

**TABLE 3:** Death censored graft and patient survival stratified by height differences.

	1 year (%)	3 years (%)	5 years (%)	Until last follow up (%)
Deceased donors				
Death censored graft survival				
R = D	94.9	90.5	86.2	76.8
R < D	95.7	91.5	87.7	78.8
R > D	93.8	88.0	82.8	73.1
Patient survival				
R = D	95.3	90.2	84.0	65.1
R < D	95.9	91.6	86.3	68.6
R > D	94.7	88.9	82.1	62.9
Living donors				
Death censored graft survival				
R = D	97.8	94.9	91.9	82.7
R < D	97.4	94.6	91.3	81.9
R > D	97.7	94.5	90.8	81.5
Patient survival				
R = D	98.4	95.8	92.4	76.7
R < D	98.4	95.7	92.4	77.3
R > D	98.3	95.3	91.8	75.9

**FIGURE 3:**
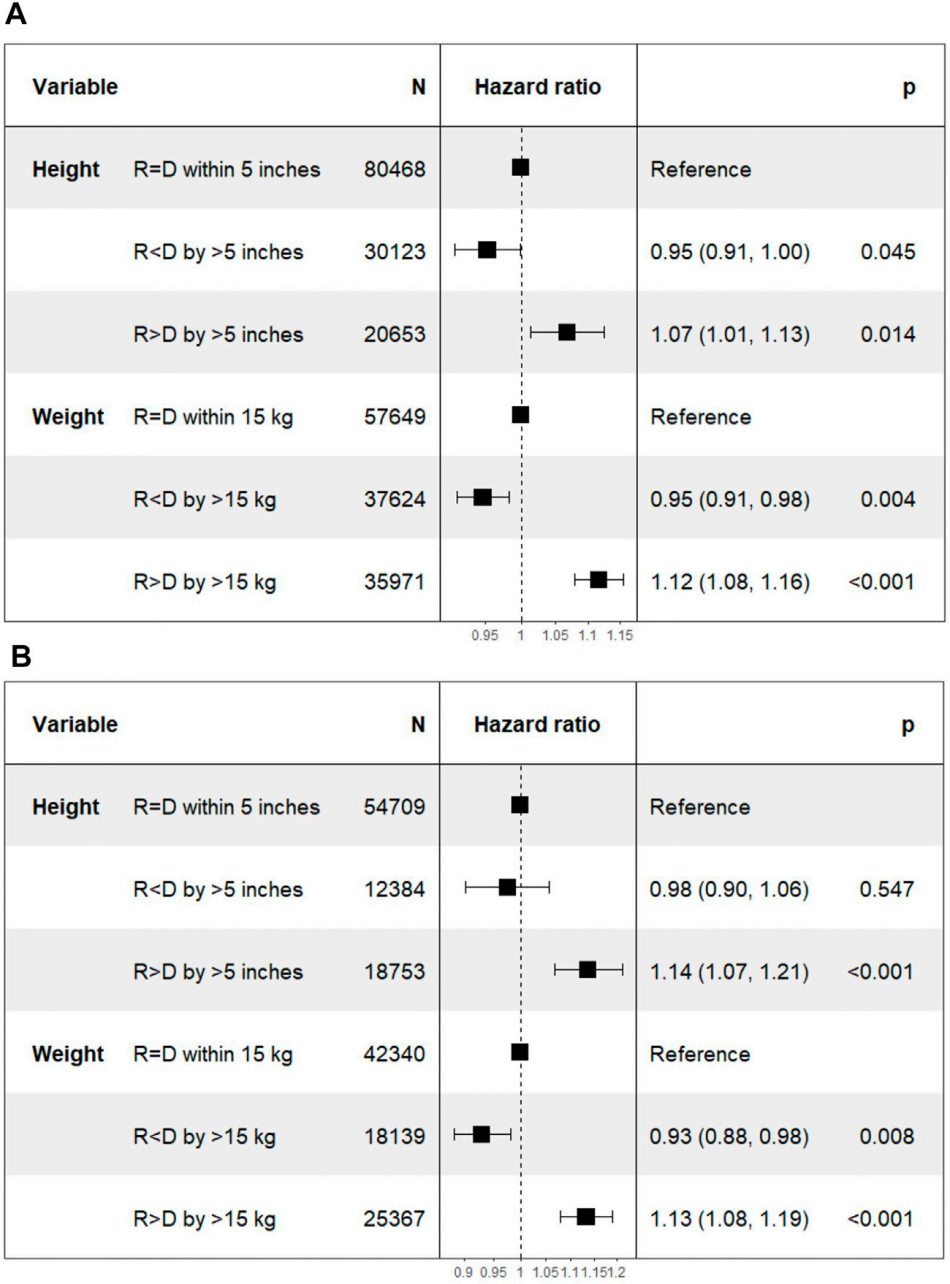
Differences in death censored graft loss with incremental differences in recipient and donor heights. [Height of recipient (R) shorter than (<) or taller than (>) donor (D) by more than 5 inches, or within 5 inches (=)]. **(A)** Deceased donor kidney transplant. **(B)** Living donor kidney transplant.

On Cox multivariate regression analysis using recipient-donor ΔHeight and ΔWeight along with other covariates as discussed above, the HR [95% confidence interval (CI)] of Recipient < Donor was lower at 0.95 (0.91–1.00; *p* = 0.05) and that of Recipient > Donor was higher at 1.07 (1.01–1.13; *p* = 0.01) compared to the reference group of Recipient = Donor n the DD cohort. The HR (95% CI) for the two extremes of ΔWeight were similarly lower for Recipient < Donor group at 0.95 (0.91–0.98; *p* = 0.004) and higher for Recipient > Donor group at 1.12 (1.08–1.16; *p* < 0.001) compared to the reference group of Recipient = Donor (Figure 3A).

##### Living Donor Kidney Transplants

On Kaplan Meier analysis, there was a statistically significant difference in DCGL with incremental degrees of ΔHeight. The Recipient < Donor group had the lowest rates of DCGL followed by Recipient = Donor and Recipient > Donor groups (*p* < 0.001). (Figure 2B).

The 1, 3, and 5 years graft survival for LD transplant recipients were 97.4, 94.6, and 91.3% respectively for Recipient < Donor group, 97.8, 94.9, and 91.9% for Recipient = Donor group and 97.7, 94.5, and 90.8% for Recipient > Donor group. At last follow up, the graft survival rates were 81.9, 82.7, and 81.5% respectively. The absolute difference in graft survival rates between the two extremes of Recipient < Donor and Recipient > Donor groups was 0.4% (Table 3).

The HR (95% CI) of DCGL in Recipient < Donor group was lower at 0.98 (0.90–1.06; *p* = 0.55) for Recipient < Donor group and higher at 1.14 (1.07–1.21; *p* < 0.001) in Recipient > Donor group compared to Recipient = Donor group. In the ΔWeight categories, the HR (95% CI) of DCGL was lower at 0.93 (0.88–0.98; *p* = 0.008) in Recipient < Donor group and higher at 1.13 (1.08–1.19; *p* < 0.001) in Recipient > Donor group compared to the Recipient = Donor group (Figure 3B).

The model with height differences as a covariate performed better than the model without height differences with lower AIC scores for both DD and LD transplants (*p* value with likelihood ratio test <0.001) (Supplementary Table S1).

### Secondary Outcomes

#### Mortality

##### Deceased Donor Kidney Transplants

**FIGURE 4:**
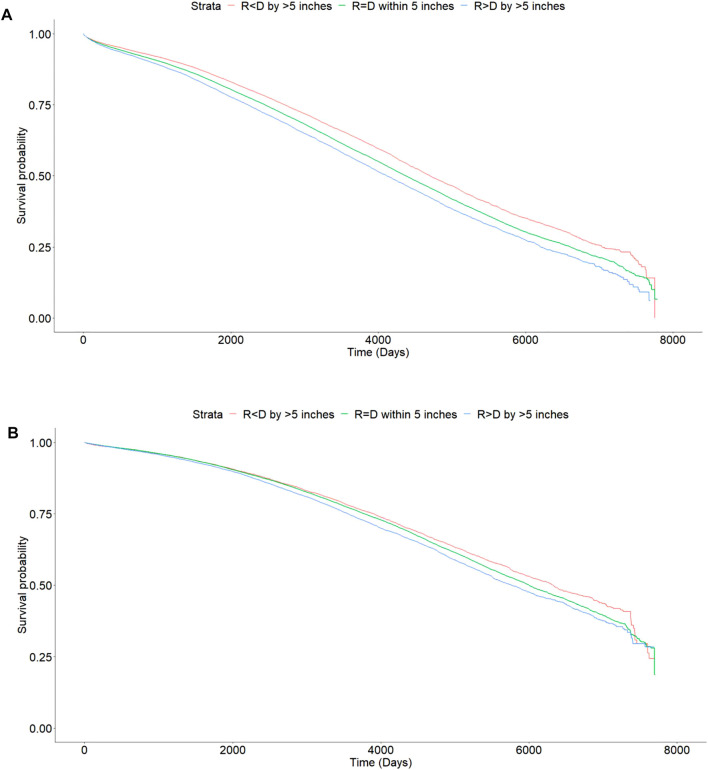
Kaplan Meier curves showing differences in mortality with incremental difference between recipient and donor heights [Height of recipient (R) shorter than (<) or taller than (>) donor (D) by more than 5 inches, or within 5 inches (=)]. **(A)** Deceased donor transplants, *p* < 0.001. **(B)** Living donor transplants, *p* < 0.001.

On Kaplan Meier analysis, there was a statistically significant difference in patient survival between the different categories of ΔHeight (*p* value < 0.001) (Figure 4A).

The 1, 3, and 5 years patient survival for DD transplant recipients were 95.9, 91.6, and 86.3% for Recipient < Donor group, 95.3, 90.2, and 84.0% for Recipient = Donor group, 94.7, 88.9, and 82.1% for Recipient > Donor group respectively. At last follow up, the patient survival rates were 68.6, 65.1, and 62.9% respectively. The absolute difference in patient survival rates between the two extremes of Recipient < Donor and Recipient > Donor groups was 5.7% (Table 3).

**FIGURE 5:**
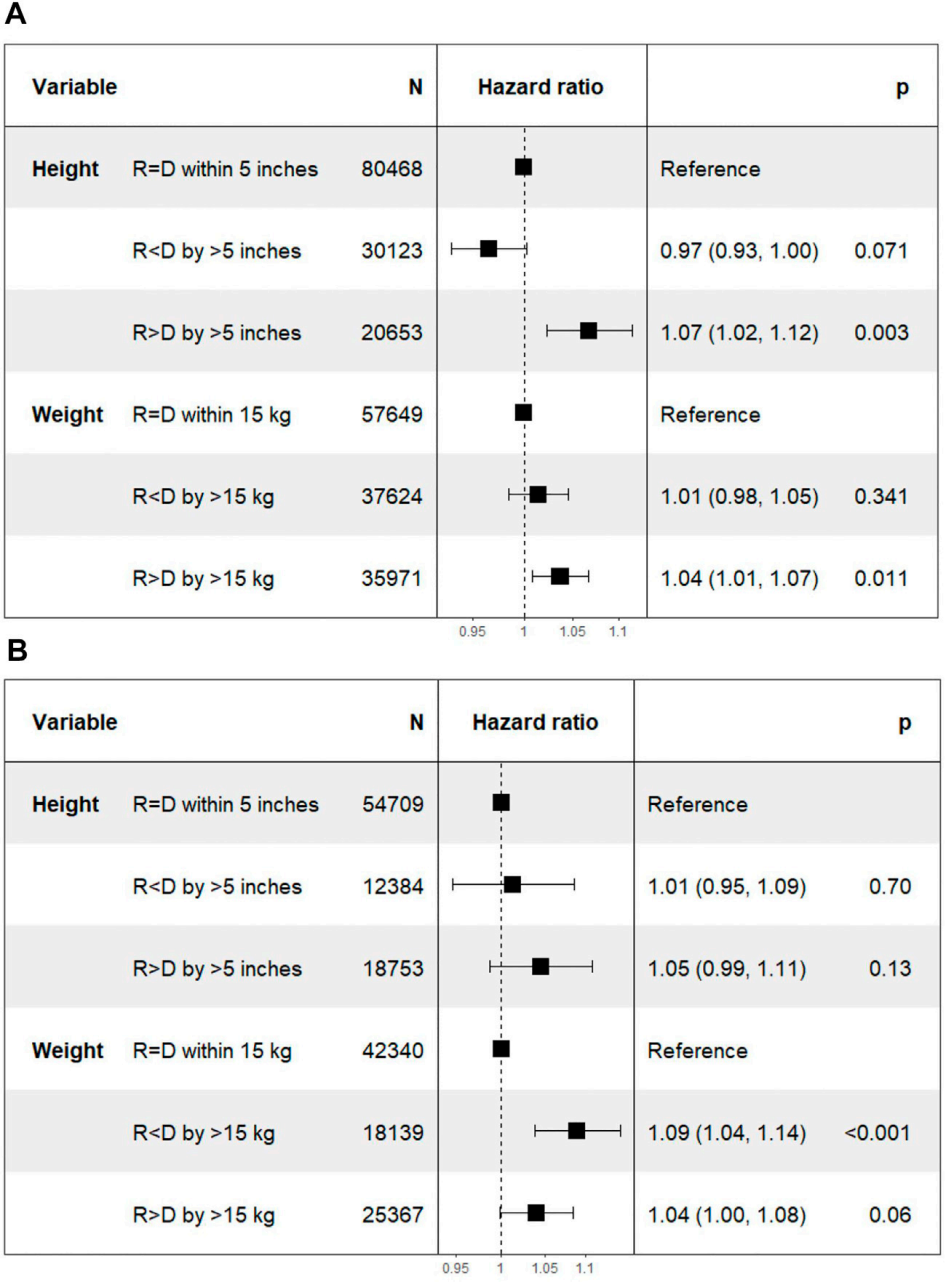
Differences in mortality with incremental differences in recipient and donor height and weight. [Height of recipient (R) shorter than (<) or taller than (>) donor (D) by more than 5 inches, or within 5 inches (=)]. **(A)** Deceased donor transplant. **(B)** Living donor transplant.

On Cox multivariate analysis using recipient-donor ΔHeight and ΔWeight along with other covariates as discussed above, there was a statistically significant higher HR of mortality in Recipient > Donor group for both ΔHeight [1.07 (1.02–1.12); *p* = 0.003] and ΔWeight [1.04 (1.01–1.07); *p* = 0.01] categories using Recipient = Donor as the reference category in the DD cohort. However, the HR for Recipient < Donor were not significant for either ΔHeight [0.97 (0.93–1.00); *p* = 0.07] or ΔWeight [1.01 (0.98–1.05); *p* = 0.34] categories (Figure 5A).

##### Living Donor Transplants

On Kaplan Meier analysis, there was a statistically significant difference in patient survival between the different categories of ΔHeight (*p* value < 0.001) (Figure 4B).

The 1, 3, and 5 years patient survival for LD transplant recipients were 98.4, 95.7, and 92.4% for Recipient < Donor group, 98.4, 95.8, and 92.4% for Recipient = Donor group, and 98.3, 95.3, and 91.8% respectively for Recipient > Donor group. At last follow up, the patient survival rates were 77.3, 76.7, and 75.9% respectively. The absolute difference in patient survival rates between the two extremes of Recipient < Donor and Recipient > Donor groups was 1.4% (Table 3).

The HR (95% CI) of mortality was 1.01 (0.95–1.09; *p* = 0.7) in Recipient < Donor group and 1.05 (0.99–1.11; *p* = 0.13) in Recipient > Donor group. Similarly, in the weight categories, the HR (95% CI) was 1.09 (1.04–1.14; *p* < 0.001) in Recipient < Donor and 1.04 (1.00–1.08; *p* = 0.06) in Recipient > Donor group (Figure 5B).

The model with height differences performed better than the one without height differences with lower AIC scores for both DD and LD transplants (*p* value with likelihood ratio test <0.001) (Supplementary Table S1).

#### Delayed Graft Function

##### Deceased Donor Kidney Transplants

In DD transplant recipients, multivariate logistic regression showed no statistical difference in DGF in Recipient < Donor [1.04 (0.99–1.1); *p* = 0.1] and Recipient > Donor [1.01 (0.95–1.08; *p* = 0.7] groups stratified by ΔHeight. However, there was a higher HR of DGF with both Recipient < Donor [1.07 (1.03–1.12); *p* < 0.001] and Recipient > Donor [1.20 (1.16–1.25); *p* < 0.001] groups compared to the Recipient = Donor group stratified by ΔWeight.

##### Living Donor Kidney Transplants

**FIGURE 6:**
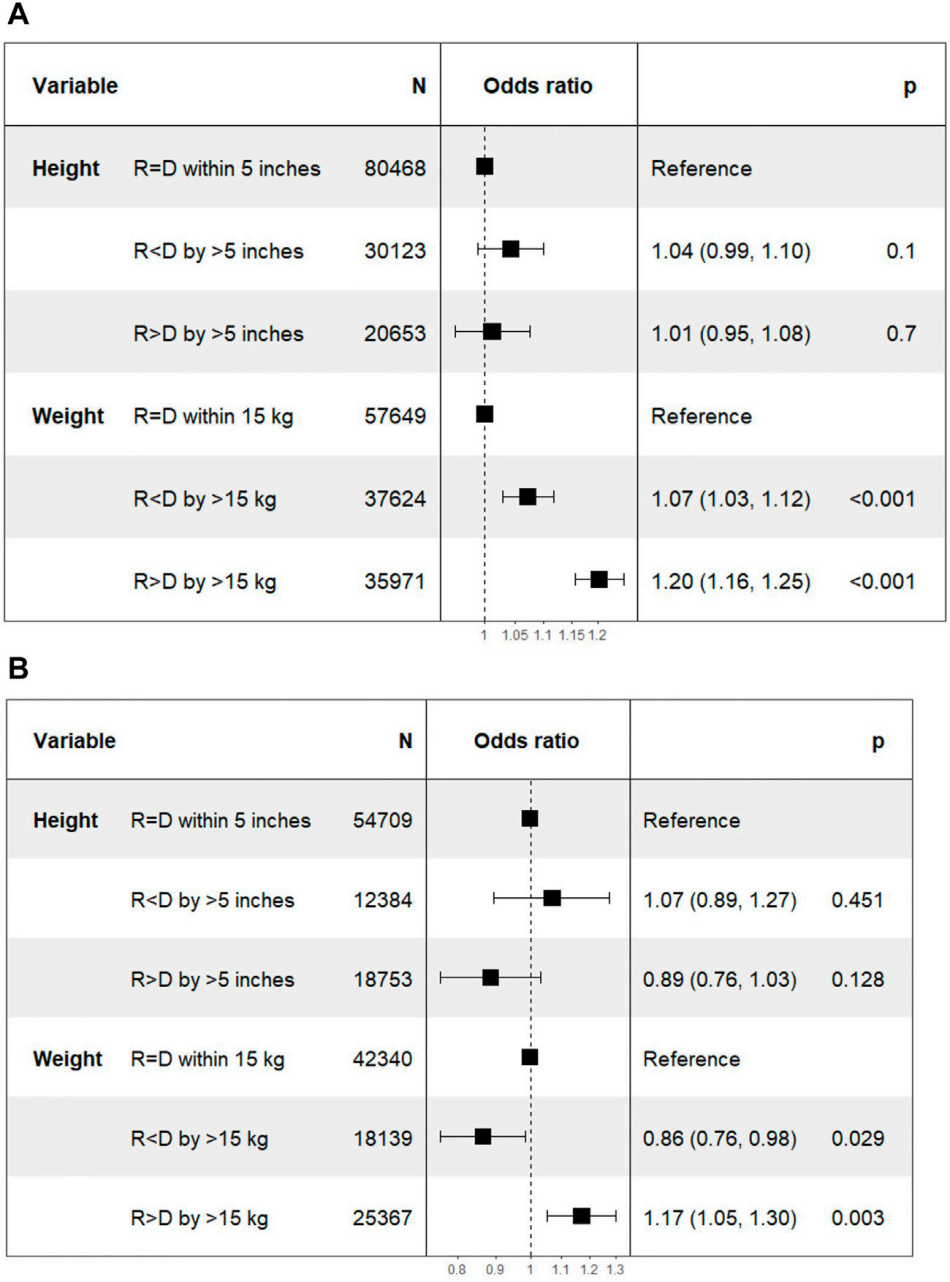
Differences in delayed graft function with incremental differences in recipient and donor height and weight. [Height of recipient (R) shorter than (<) or taller than (>) donor (D) by more than 5 inches, or within 5 inches (=)]. **(A)** Deceased donor transplants. **(B)** Living donor transplants.

In LD transplant recipients, the HR (95% CI) of DGF in Recipient < Donor and Recipient > Donor groups were 1.07 (0.89–1.27; *p* = 0.45) and 0.89 (0.76–1.03; *p* = 0.13) for ΔHeight categories. The corresponding HR for ΔWeight categories were 0.86 (0.76–0.98; *p* = 0.03) and 1.17 (1.05–1.30; *p* = 0.003) respectively (Figure 6).

#### Overall Graft Loss

##### Deceased Donor Kidney Transplants

In the deceased donor cohort, there was a statistically significant lower HR in the Recipient < Donor category of 0.97 (0.94–1; *p* = 0.04) and higher HR in the Recipient > Donor category of 1.06 (1.02–1.1; *p* = 0.002).

##### Living Donor Kidney Transplants

In the living donor cohort, there was an HR of 1.00 (0.95–1.06; *p* = 0.88) in the Recipient < Donor category and a higher HR of 1.06 (1.01–1.11; *p* = 0.02) in the Recipient > Donor group (Supplementary Figure S5).

#### Death With a Functioning Graft

##### Deceased Donor Kidney Transplants

In the deceased donor cohort, there was a lower HR of 0.97 (0.93–1.01; *p* = 0.19) in the Recipient < Donor category and a higher HR of 1.06 (1.01–1.12; *p* = 0.02) in the Recipient > Donor category.

##### Living Donor Kidney Transplants

In living donor cohort, there was a higher HR of 1.02 (0.94–1.11; *p* = 0.6) and 1.01 (0.95–1.08; *p* = 0.7) in Recipient < Donor and Recipient > Donor categories respectively (Supplementary Figure S6).

#### Interaction Between Differences in Donor-Recipient Height and Weight

Model fit improved with the addition of height differences and weight differences separately. Including both height and weight differences resulted in the best model fit. However, the inclusion of an interaction term between height difference and weight difference did not improve the model fit as evidenced by higher AIC scores and statistically non-significant *p* values (Supplementary Table S1).

#### Donor-Recipient Gender Combination

**FIGURE 7:**
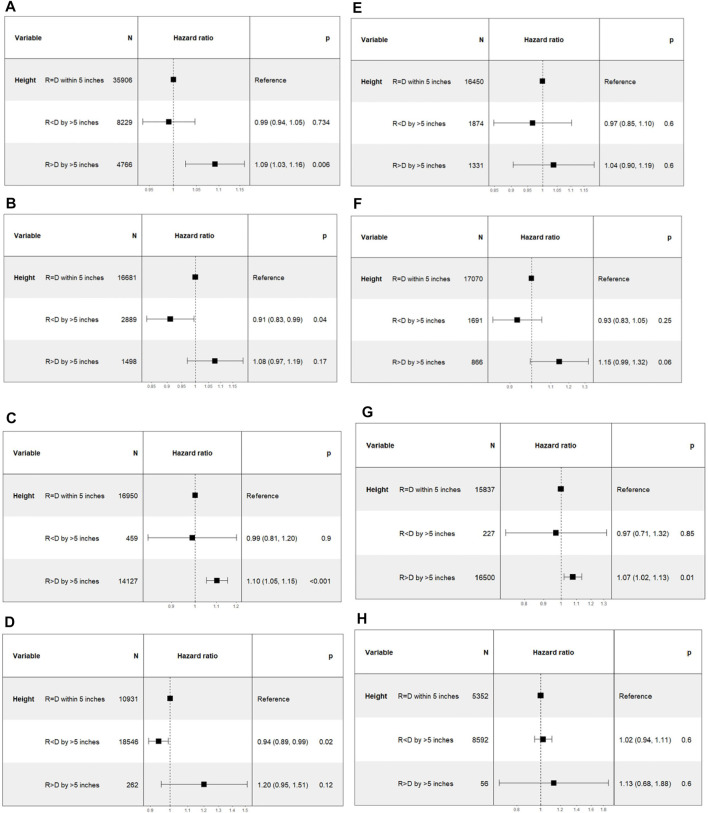
Differences in death censored graft loss with incremental differences in recipient and donor heights, categorized on the basis of gender differences between the donor-recipient pairs. [Height of recipient (R) shorter than (<) or taller than (>) donor (D) by more than 5 inches, or within 5 inches (=)]. **(A)** Deceased donor male to male transplant. **(B)** Deceased donor female to female transplant. **(C)** Deceased donor female to male transplants. **(D)** Deceased donor male to female transplant. **(E)** Living donor male to male transplants. **(F)** Living donor female to female transplant. **(G)** Living donor female to male transplant. **(H)** Living donor male to female transplant.

As a sub-group analysis, the DD and LD cohorts were divided into four categories of male to male, female to female, male to female and female to male transplants. Within each of these categories, a consistent trend of a lower hazard ratio for Recipient < Donor and a higher hazard ratio for Recipient > Donor was seen in both DD and LD transplants—among DD, male to male transplants Recipient > Donor group [n = 4766; 1.09 (1.03–1.16); *p* = 0.006], female to female transplants Recipient < Donor group [n = 2889; 0.91 (0.83–0.99); *p* = 0.04], female to male transplants Recipient > Donor group [n = 14127; 1.10 (1.05–1.15); *p* < 0.001], male to female transplants Recipient < Donor group [n = 18546; 0.94 (0.89–0.99); *p* = 0.02], and among LD, female to male transplants Recipient > Donor group [n = 16,500; 1.07 (1.02–1.13); *p* = 0.01] (Figure 7).

Inclusion of height and gender pair differences led to better model fit but the inclusion of an interaction term between height differences and gender pair differences did not improve the model fit as evidenced by higher AIC scores (Supplementary Table S1).

## Discussion

Size mismatch between the recipient and donor is known to influence transplant outcomes. However, the impact of height mismatch specifically is not known. In this analysis of a large database of deceased and living donor kidney transplant patients from SRTR, we found that height mismatch between the recipient and donor is an independent factor predicting kidney transplant outcomes.

Prior studies have evaluated size mismatch between the recipient and donor on the basis of body surface area (BSA), body mass index (BMI), and weight (2,3,8,9). These parameters have been used as surrogates for discrepancies in kidney size and/or nephron mass in the recipient-donor pair. We found that there is a poor correlation between an individual’s height and weight. At a given weight, there was a wide variation of heights in the population (Supplementary Figures S3, S4). Similarly, despite the derivation of BSA from height and weight of the individual, we only found a modest correlation of BSA with height (r = 0.69 for DD, r = 0.7 for LD), but a high correlation with weight (r = 0.98 for DD, r = 0.98 for LD).

Height discrepancy in the recipient-donor pairs has not been studied rigorously as a predictor of outcomes in kidney transplant patients without being included in a composite measure such as BSA or BMI. In a study done by Vinson et al, their risk prediction model showed a statistically significant lower hazard ratio with increasing donor-recipient height difference in DD transplants [0.726 (0.664–0.794)] (10). Our study had similar findings, with a more robust categorization of height discrepancies, and included DD and LD cohorts separately. Donor-recipient height ratios have also been included in a kidney graft survival calculator that showed a lower hazard ratio for a height ratio >1.06 [0.94 (0.91–0.98)] and a higher hazard ratio for a height ratio <0.94 [1.05 (1.02–1.09)] (11).

It is not clear from the literature which anthropometric measurement is the best surrogate for nephron mass. One of the predictors of nephron mass is birth weight and length (1,4–6), which has a strong association with adult height (12). It is well known that nephron endowment at the time of birth is final and any deficit due to pre-maturity exposes the individual to a higher likelihood of developing kidney disease during their lifetime, due to the increased demands placed on the lower number of nephrons. Although these do influence adult weight as well, it is prone to be modified by an individual’s lifestyle such as dietary habits and physical activity, and fluid balance. Higher weight has been shown to be associated with larger nephron size, but not necessarily with a higher number of nephrons (4). A larger nephron size may be a reflection of increased metabolic demand on a limited number of nephrons. Higher recipient BMI has been shown to be associated with increased morbidity and graft loss after kidney transplantation (13,14).

In their population based study from the UK Transplant Registry, Arshad et al did not find any differences in DGF or DCGL due to donor-recipient weight differences but they did find increased mortality in patients receiving kidneys from donors whose weights were over 25% of recipient weight (2). Miller et al found that a concurrent mismatch in donor-recipient weight and donor-recipient sex was associated with a higher risk of DCGL (8). This finding could be secondary to a higher weight in the donor being a reflection of other co-morbidities that accompany obesity such as diabetes mellitus and hypertension. In addition, obesity could lead to hyperfiltration injury in the donor kidney that would likely not be the case in non-obese donors. Lepeytre et al reported that the effect of donor-recipient size mismatch based on their BSA on long-term transplant outcomes is modulated by the recipient and donor age in their population based study from SRTR (3). This is not surprising as a serial decline in the number of nephrons with age parallels the progressive decline in glomerular filtration rate with age (4,15,16). Instead of using a composite measure such as BSA or BMI in prior studies, we evaluated the individual components of these measures to reconcile the discrepant results in prior studies.

Gender differences are also an important factor to consider when assessing size mismatch (8,9,17,18). We found that the influence of height discrepancies persisted within different combinations of donor-recipient genders in a predictable pattern just as in the overall cohort of DD and LD transplants. In subgroups that contained a large number of patients, the trends were statistically significant. However, we suspect some categories failed to show statistical significance due to the relatively fewer number of patients in the category. Regardless, the trends show that Recipient < Donor have better outcomes and Recipient > Donor have poorer outcomes compared to the Recipient = Donor group (Figure 7). Inclusion of height differences to a model including gender pair differences improved the model fit for DCGL and mortality but the interaction effect was not significant, suggesting that the height differences do not impact the outcomes differently among those with different donor-recipient gender combinations.

Our study has several strengths. It is based on a large database of transplant populations from SRTR in the modern era of tacrolimus-based maintenance immunosuppression and transplant care. Our study utilizes height as an anthropometric measure to assess size mismatch, which likely correlates best with nephron mass and is less likely to be influenced by an individual’s lifestyle choices. The use of height mismatch is also simpler to use compared to other composite anthropometric measures such as BMI and BSA. Although donor height is incorporated into the allocation system as a part of the KDPI score, height mismatch between the donor-recipient pair may be a more important factor to consider in terms of transplant outcomes. Our study also incorporates analyses of multiple models with interactions between donor-recipient height differences and differences in weight and gender to determine the differential impact of these co-occurring donor-recipient mismatches.

The results of this population-based study must be interpreted within the limitations of the design of these studies. While we made efforts to minimize bias by incorporating multiple covariates that could influence transplant outcomes, it is not possible to include all the variables. We acknowledge that there could be residual confounding resulting from variables that could not be incorporated into our analysis. We could not assess the degree of proteinuria in the patients in the different subgroups as this information was not available in the database. This would have allowed us to see the impact of height mismatch on downstream physiologic effects such as progressive glomerular sclerosis and resultant urinary protein excretion. The results of our study are based on data from the US population and may not be generalizable to other populations.

In conclusion, our study finds that transplantation of kidneys from individuals of shorter stature into taller recipients leads to worse transplant outcomes. This effect appeared more pronounced in deceased donors than in living donors. This information may be used while counseling living donors and their recipients that height mismatch may not have a major influence in determining post-transplant outcomes, especially when multiple donors are available. The quality of a living donor kidney and recipient comorbidities likely supersede the influence of height mismatch in living donor transplantation. Size mismatch in the donor-recipient pair based on discrepancies in their heights may be more reliable compared to other anthropometric measures in determining post-transplant outcomes.

## Data Availability

The data analyzed in this study is subject to the following licenses/restrictions: A research proposal is required to be submitted to SRTR to have access to the dataset. Requests to access these datasets should be directed to https://srtr.org/requesting-srtr-data/data-requests/.

## Supplementary Material

Supplementary Figure 1 Histogram showing distribution of height differences **(A)** Deceased donor kidney transplant **(B)** Living donor kidney transplant.

Supplementary Figure 2 Histogram showing distribution of weight differences **(A)** Deceased donor kidney transplant **(B)** Living donor kidney transplant.

Supplementary Figure 3 Differences in recipient-donor heights at different categories of recipient weights for deceased donor kidney transplants.

Supplementary Figure 4 Donor-recipient pairs with height differences greater than 10 inches stratified by weight quartiles of the deceased donor transplant recipients.

Supplementary Figure 5 Cox multivariate analysis for overall graft loss with differences in donor and recipient height **(A)** Deceased donor kidney transplant **(B)** Living donor kidney transplant.

Supplementary Figure 6 Cox multivariate analysis for death with a functioning graft with differences in donor recipient height **(A)** Deceased donor kidney transplant **(B)** Living donor kidney transplant.

Supplementary Table 1 Comparison of different models with various interaction terms with differences in donor-recipient height.

## Abbreviations

AIC, Akaike Information Criterion; ANOVA, Analysis of Variance; BMI, Body Mass Index; BSA, Body Surface Area; CI, Confidence Interval; DCGL, Death censored graft loss; DD, Deceased donor kidney transplant; DGF, Delayed graft function; ESRD, End Stage Renal Disease; ΔHeight, Difference in recipient and donor height; HLA, Human Leukocyte Antigen; HR, Hazard ratio; HRSA, Health Resources and Services Administration; KDPI, Kidney Donor Profile Index; LD, Living donor kidney transplant; OPTN, Organ Procurement and Transplantation Network; PRA, Panel Reactive Antibodies; SRTR, Scientific Registry of Transplant Recipients; ΔWeight, Difference in recipient and donor weight.
