# Supplementary figures and images for: Impact of Size Matching Based on Donor-Recipient Height on Kidney Transplant Outcomes
Source: Transpl Int. 2022 Mar 18;35:10253. doi: 10.3389/ti.2022.10253 (PMC9099356; doi:10.3389/ti.2022.10253)

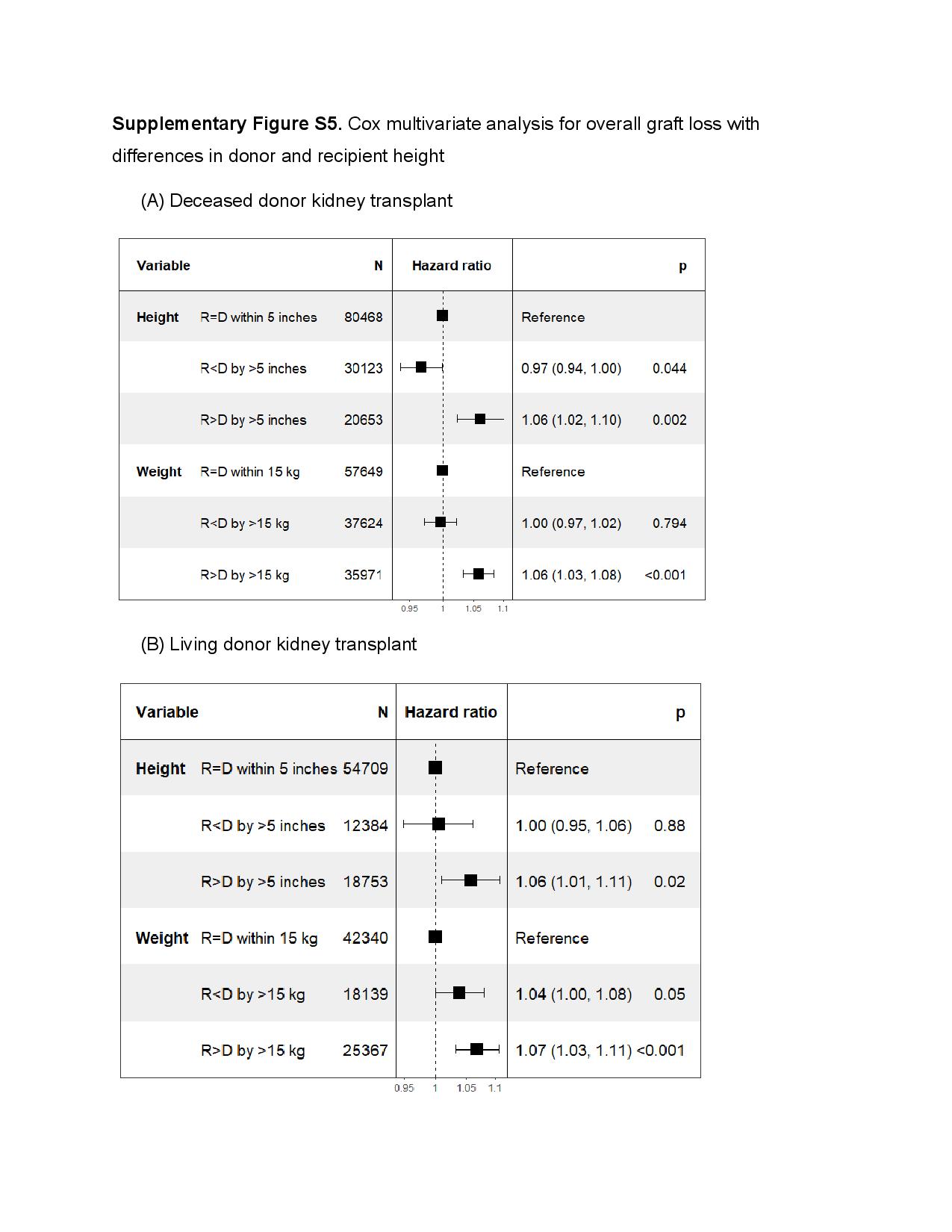

Supplement: Supplementary file 1 [file Image5.jpg]

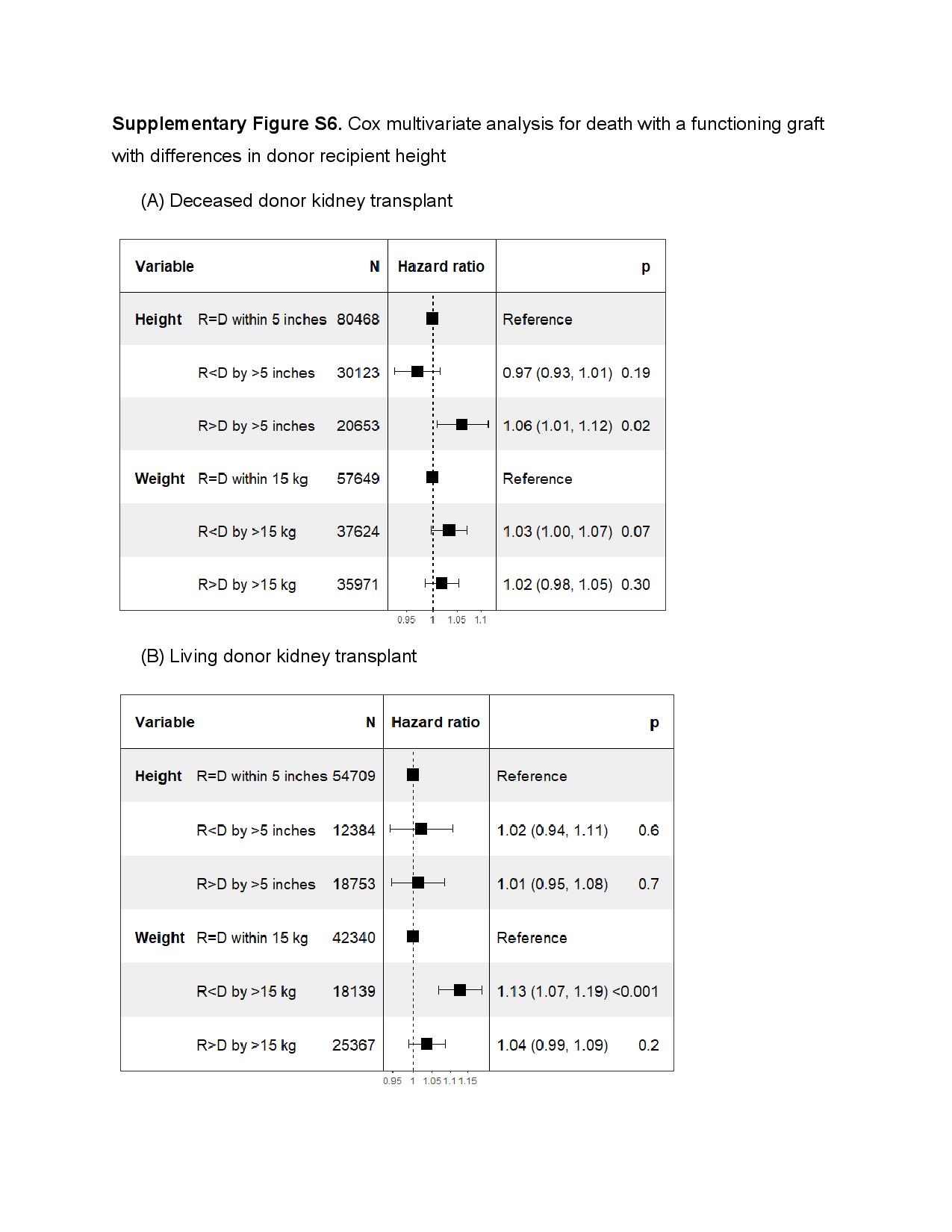

Supplement: Supplementary file 2 [file Image6.jpg]

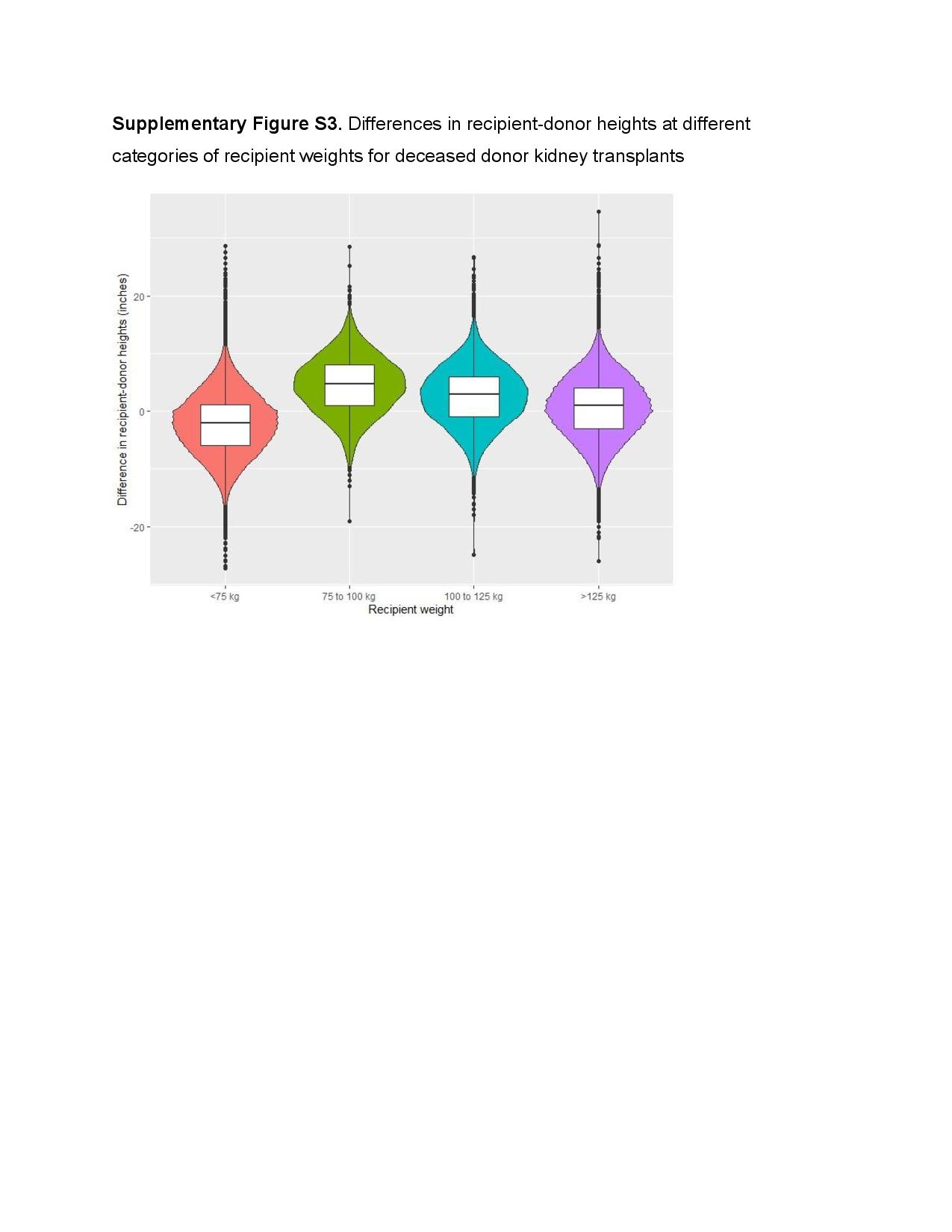

Supplement: Supplementary file 3 [file Image3.jpg]

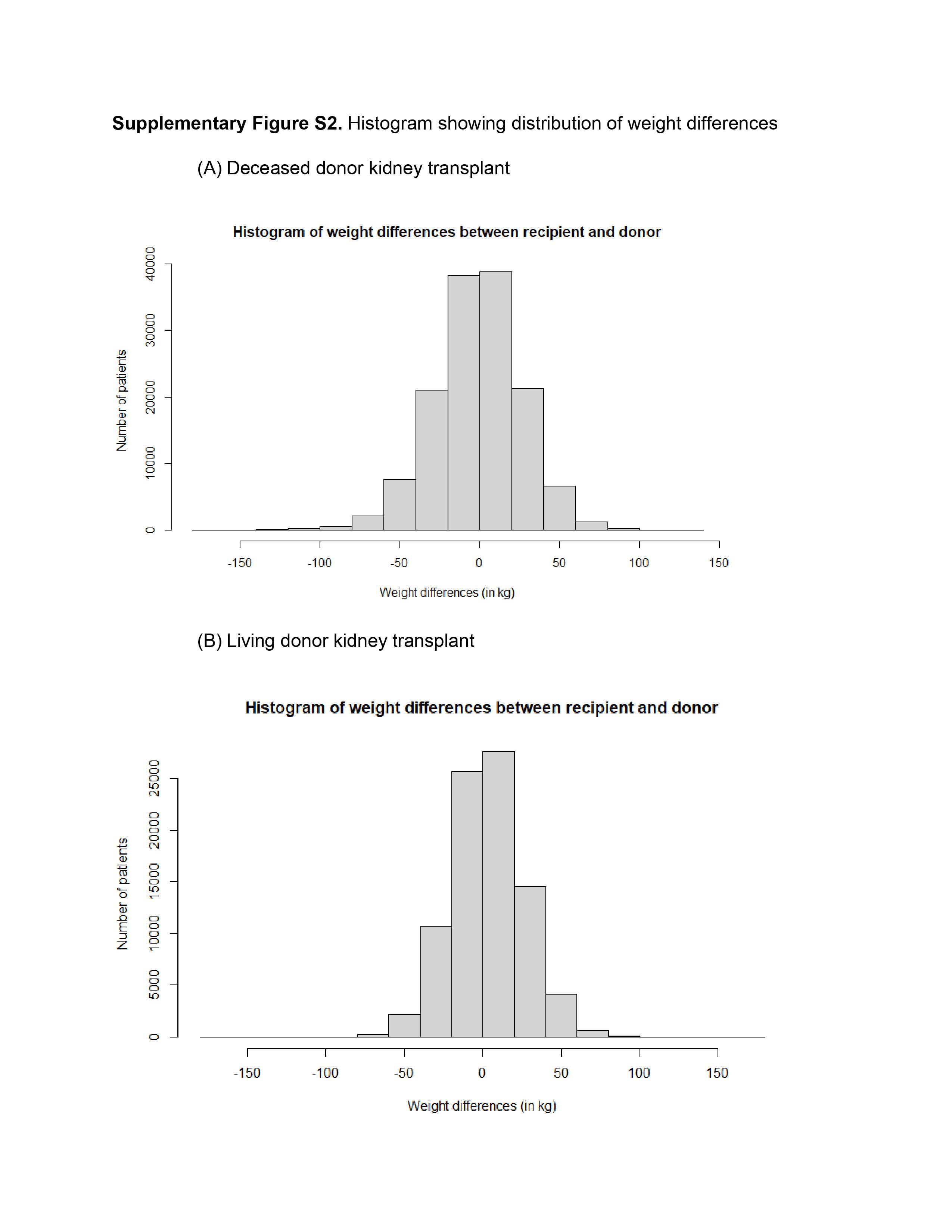

Supplement: Supplementary file 4 [file Image2.jpg]

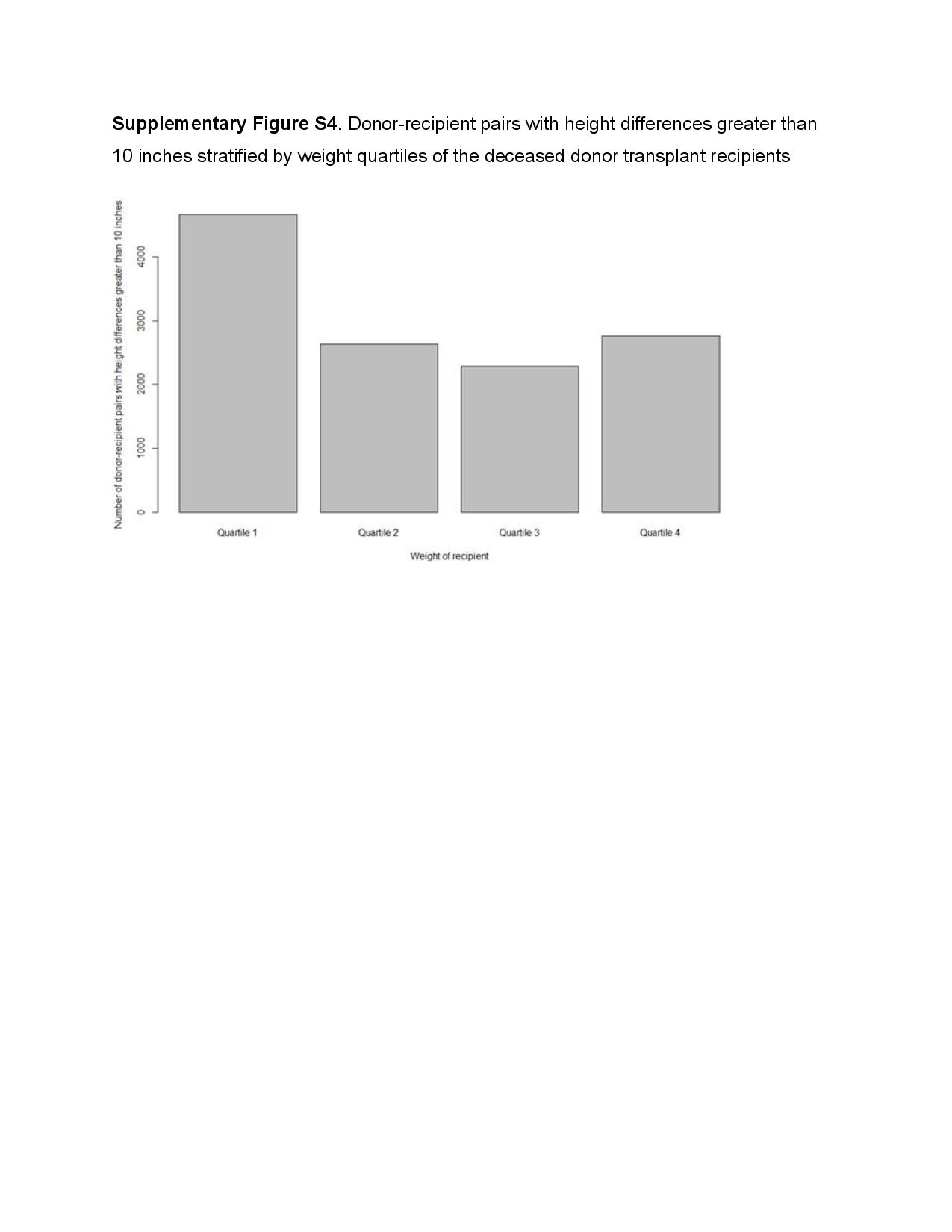

Supplement: Supplementary file 6 [file Image4.jpg]

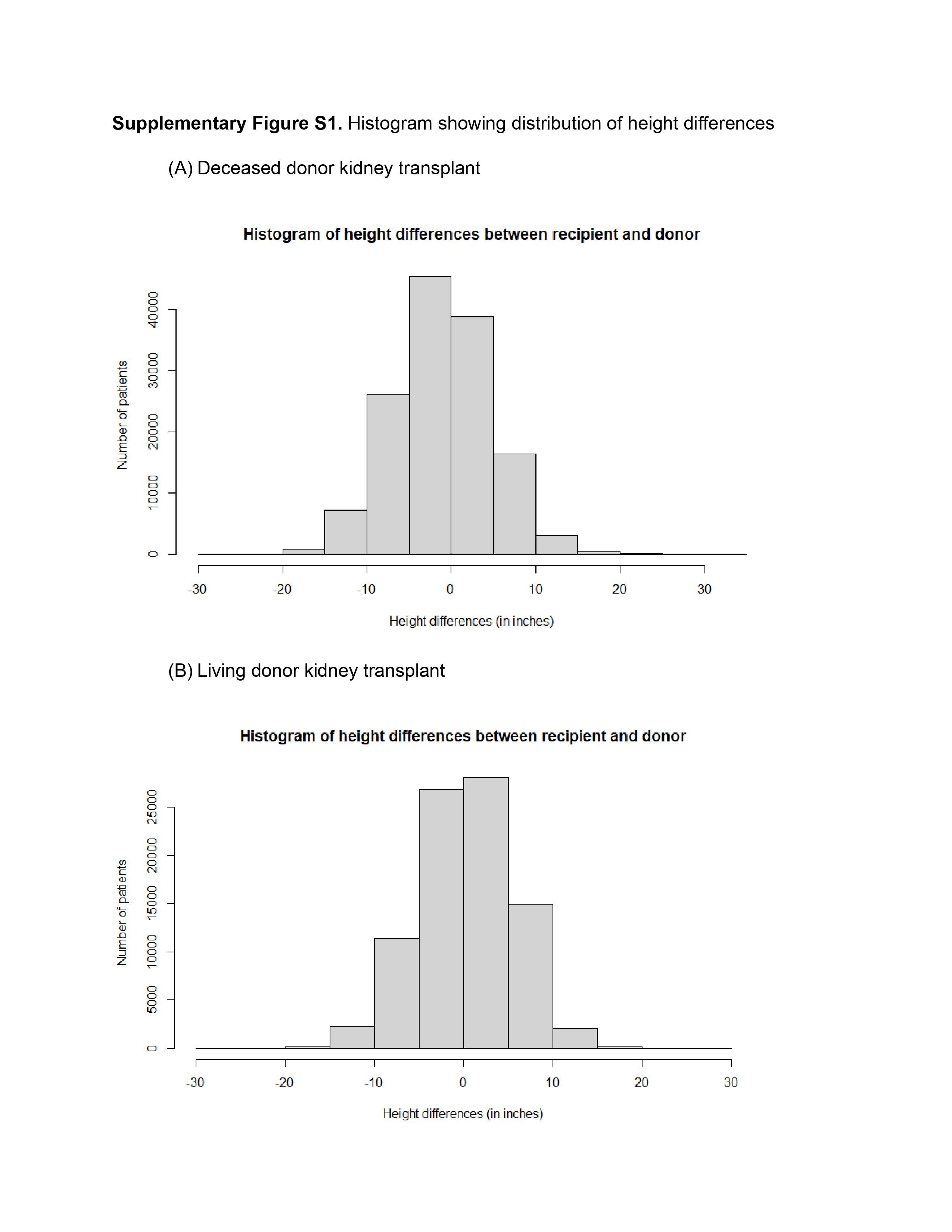

Supplement: Supplementary file 7 [file Image1.jpg]
