# Supplementary material for: Impact of Size Matching Based on Donor-Recipient Height on Kidney Transplant Outcomes
Source: Transpl Int. 2022 Mar 18;35:10253. doi: 10.3389/ti.2022.10253 (PMC9099356; doi:10.3389/ti.2022.10253)
Supplement: Supplementary file 5 [file Table1.docx]

**Supplementary Table S1.** Comparison of different models with various interaction terms with differences in donor-recipient height

| Model^#^ | AIC | Delta AIC | AIC weight | Cumulative weight | Likelihood ratio test  (*P* value)* |
| --- | --- | --- | --- | --- | --- |
| Interaction between differences in donor-recipient height and weight | | | | | |
| **Death censored graft loss in deceased donor kidney transplantation** | | | | | |
| Covariates + height difference + weight difference | 658289.9 | 0.00 | 0.66 | 0.66 | - |
| Covariates + height difference + weight difference + height difference*weight difference interaction | 658291.2 | 1.31 | 0.34 | 1.00 | 0.15 |
| Covariates + weight difference | 658324.9 | 35.02 | 0.00 | 1.00 | <0.001 |
| Covariates + height difference | 658417.1 | 127.22 | 0.00 | 1.00 | <0.001 |
| Covariates | 658504.0 | 214.09 | 0.00 | 1.00 | <0.001 |
|  | | | | | |
| **Death censored graft loss in living donor kidney transplantation** | | | | | |
| Covariates + height difference + weight difference | 319801.4 | 0.00 | 0.65 | 0.65 | - |
| Covariates + height difference + weight difference + height difference*weight difference interaction | 319802.7 | 1.28 | 0.34 | 1.00 | 0.15 |
| Covariates + weight difference | 319811.7 | 10.27 | 0.00 | 1.00 | <0.001 |
| Covariates + height difference | 319840.1 | 38.69 | 0.00 | 1.00 | <0.001 |
| Covariates | 319864.5 | 63.12 | 0.00 | 1.00 | <0.001 |
|  | | | | | |
| **Mortality in deceased donor kidney transplantation** | | | | | |
| Covariates + height difference + weight difference | 948251.7 | 0.00 | 0.87 | 0.87 | - |
| Covariates + height difference + weight difference + height difference*weight difference interaction | 948256.3 | 4.57 | 0.09 | 0.96 | 0.49 |
| Covariates + height difference | 948257.8 | 6.11 | 0.04 | 1.00 | <0.001 |
| Covariates + weight difference | 948264.8 | 13.08 | 0.00 | 1.00 | <0.001 |
| Covariates | 948277.5 | 25.82 | 0.00 | 1.00 | <0.001 |
|  | | | | | |
| **Mortality in living donor kidney transplantation** | | | | | |
| Covariates + height difference + weight difference + height difference*weight difference interaction | 408937.2 | 0.00 | 0.53 | 0.53 | - |
| Covariates + weight difference | 408938.0 | 0.83 | 0.35 | 0.88 | 0.03 |
| Covariates + height difference + weight difference | 408940.3 | 3.05 | 0.12 | 1.00 | 0.29 |
| Covariates + height difference | 408955.6 | 18.42 | 0.00 | 1.00 | <0.001 |
| Covariates | 408957.2 | 19.93 | 0.00 | 1.00 | 0.06 |
|  | | | | | |
| Interaction between differences in donor-recipient height and gender | | | | | |
| **Death censored graft loss in deceased donor kidney transplantation** | | | | | |
| Covariates + height difference + gender pair | 658286.2 | 0.00 | 0.98 | 0.98 | - |
| Covariates + height difference + gender pair + height difference*gender pair interaction | 658294.1 | 7.37 | 0.02 | 1.00 | 0.34 |
| Covariates + gender pair | 658318.7 | 32.03 | 0.00 | 1.00 | <0.001 |
| Covariates | 658334.3 | 47.58 | 0.00 | 1.00 | <0.001 |
|  | | | | | |
| **Death censored graft loss in living donor kidney transplantation** | | | | | |
| Covariates + height difference + gender pair | 319795.7 | 0.0 | 0.85 | 0.85 | - |
| Covariates | 319800.7 | 4.95 | 0.07 | 0.92 | 0.82 |
| Covariates + gender pair | 319800.7 | 4.95 | 0.08 | 0.99 | 0.01 |
| Covariates + height difference + gender pair difference + height difference*gender pair interaction | 319804.8 | 9.1 | 0.01 | 1.00 | <0.001 |
|  |  |  |  |  |  |
| **Mortality in deceased donor kidney transplantation** | | | | | |
| Covariates + height difference + gender pair difference | 948251.7 | 0.00 | 0.97 | 0.97 | - |
| Covariates + height difference + gender pair difference + height difference*gender pair difference interaction | 948258.4 | 6.72 | 0.03 | 1.00 | 0.13 |
| Covariates | 948264.8 | 13.08 | 0.00 | 1.00 | <0.001 |
| Covariates + gender pair | 948270.5 | 18.83 | 0.00 | 1.00 | 0.61 |
|  |  |  |  |  |  |
| **Mortality in living donor kidney transplantation** | | | | | |
| Covariates + gender pair | 408939.8 | 0.00 | 0.36 | 0.36 | - |
| Covariates | 408939.8 | 0.00 | 0.36 | 0.71 | 0.73 |
| Covariates + height difference + gender pair | 408940.3 | 0.45 | 0.28 | 1.00 | 0.17 |
| Covariates + height difference + gender pair difference + height difference*gender pair interaction | 408948.7 | 8.83 | 0.00 | 1.00 | <0.001 |

#Common covariates used in all models are not shown

*Chi-squared test for likelihood ratio was analyzed
